# Building Resilient Healthcare Teams: Insights from Analogy to the Social Biology of Ants, Honey Bees and Other Social Insects

**DOI:** 10.5334/pme.1051

**Published:** 2023-06-26

**Authors:** Sayra Cristancho, Graham Thompson

**Affiliations:** 1 Dept. of Surgery and Centre for Education Research & Innovation (CERI), The University of Western Ontario, Canada; 2 Department of Biology, Faculty of Science, The University of Western Ontario, Canada

## Abstract

The resilience of a healthcare system hinges on the adaptability of its teams. Thus far, healthcare teams have relied on well-defined scopes of practice to fulfill their safety mandate. While this feature has proven effective when dealing with stable situations, when it comes to disruptive events, healthcare teams find themselves navigating a fine balance between safety and resilience. Therefore, a better understanding of how the safety vs resilience trade-off varies under different circumstances is necessary if we are to promote and better train for resilience in modern healthcare teams. In this paper, we aim to bring awareness to the sociobiology analogy that healthcare teams might find useful during moments when safety and adaptability have the potential to conflict. Three principles underpin the sociobiology analogy: communication, decentralization, and plasticity. Of particular interest in this paper is plasticity whereby swapping roles or tasks becomes an adaptive, rather than a maladaptive, response teams could embrace when facing disruptive situations. While plasticity has naturally evolved in social insects, infusing plasticity in healthcare teams requires intentional training. Inspired by the sociobiology analogy, such training must value the ability: a) to read each other’s cues and miscues, b) to step aside when others had the necessary skills, even if outside their scope, c) to deviate from protocols, and d) to foster cross-training. If the goal is to increase a team’s behavioural flexibility and boost their resilience, this training mindset should become second nature.

## Introduction

Nature is exceptionally agile and resilient. It contains striking examples of collective work in group-living animals, as seen in the highly coordinated behaviour of social insects (e.g., ants, honey bees and other social bees, termites), and in some highly social species of fish and birds that, like social insects, have evolved coordinated behaviour that emerges without central control [1]. Human teams can likewise behave in a coordinated manner, but they often rely on key individuals that oversee and manage the team’s collective effort. Yet, this managerial approach is not obviously superior or any more effective than the unmanaged responses we see in nature, particularly in moments of disruption. The difference though lies on what guides collective work. In social insects and other non-human societies, the collective responds to changes in the environment and to each other to keep a colony alive and reproducing for as long as possible; selection has therefore promoted collective rather than individual function [2]. The implication is not to say that human teams should supress individual needs or leadership but rather to emphasize through a non-human analogy the possibility for new creative strategies that instill a collective perspective in our teams. Therefore, we ask, what can healthcare teams learn from the sociobiology analogy?

Creative analogies are useful to inspire new insights, comparisons, and new ways of thinking about issues. For instance, in medical education, researchers have used analogies such as the beetle-in-a-box that illustrates how private experiences are inaccessible to the public [3], or the notion of philosophical gardening that exemplifies the ways in which we should relate to those we care for [4]. These analogies are useful because they productively problematize the difficult task of measuring deeply personal experiences such as the development of a professional identity. Aviation and other high-reliability industries provide yet more analogies that have been influential in advancing the highly valued safety mandate in healthcare [5]. These analogies do break down however where the industries differ. Take the notion of resilience – the capacity of a system to absorb disruption [6]. While both industries demand resilience to ensure sustainability, the approach to it might look different. For the most part, aviation tends to embrace “engineering resilience” where the goal is to return, as much as possible, to the same state the situation was in before the disruption. In healthcare, situations rarely have a single stable state. Therefore, “ecological resilience” might be a more realistic goal – that is the idea that a situation has multiple stable states that depend on circumstance and that the system can move between when disrupted [7]. Thus aviation as analogy might fall short in healthcare situations where disruptive situations may regularly prompt a departure from procedural norms. Using an ecological lens, as it has been already advocated in medical education [8,9], this is the context where the sociobiology analogy provides an opportunity to reconsider, complement and inspire new ways of training for resilience in healthcare teams.

## Learning from sociobiology – the key principles

What does the sociobiology analogy offer that others don’t? The main idea is that collective action is achieved via three key principles: communication, decentralization, and plasticity.

In social insects, same as in humans, communication is multi-modal. For example, humans use spoken language, unspoken gestures and expressions, and even use objects as a form of trace communication whereby signals are conveyed indirectly through the use and placement of inanimate things. Likewise, social insects can use acoustics, vibration and touch among other modes of communication to convey basic information like alarm, presence, hunger and interest among nestmates and other conspecifics [10]. Ants, termites and social bees may likewise use chemically complex pheromonal signals that can function to recruit others into mating aggregations, onto profitable food sources or towards new nesting sites [11]. We have demonstrated that healthcare teams regularly use multiple forms of communication, including trace objects and other forms of indirect communication [12]. Ophthalmoscopes, bedside charts, instrument trays, syringes, among other objects are, in a way, like pheromones; they can be used to passively communicate messages to team mates, much like how ants and other social insects can communicate with their nestmates. What’s important about the communication principle from sociobiology is that it brings awareness to unspoken and no-contact forms of communication that we may not be consciously aware of and that might serve as an effective complement to team communication practices.

Because ants operate on a vast territory for their size, relying on the central control of a single decision-maker to coordinate decisions, like where to find food, would prove inefficient. To decentralize decisions is therefore an adaptation that allows individuals to respond to disruptions in their environment without having to gain permission or adhere to rigid pre-assigned tasks. Social insects can therefore use a decentralized and distributed leadership [13]. For instance, ant colonies may arrange themselves into sub-teams of, say, one leader and two specialists. The specialists provide local information to the leader who then integrates it to guide behaviour of the other two. As such their individual roles become coordinated into a group effort that is immediately responsive to changes and that no one or even three individuals could achieve on their own. A comparable type of dispersed leadership is evident in health care teams that work outside formal hospital contexts to provide care at the point of injury and to facilitate patient transport [14].

The final principle is plasticity, which hinges on the concept of division of labour where social insects organize in groups, known as castes, to perform tasks [14]. An ideal division of labour would consist of specialists for each task and subtask. However, given the unpredictable environments that social insects live in, the number of specialists available at a moment’s time is usually constrained. What do they do then to maximize their resilience? Importantly, they are sufficiently plastic to switch from one task or role to another [13]. Therefore, when the environment is predictable and bountiful, roles are well-defined. But when the environment is disrupted, colonies quickly adjust task allocation to better match the circumstance. Might human health care teams benefit from increased plasticity?

## The plasticity conundrum

In health care, we have struggled with the idea that individuals working within teams could act as specialists and generalists, depending on the circumstance [15,16]. Therefore, swapping or interchanging tasks, roles or people is uncomfortable because of the strong value we put on specialization and well-defined scopes of practice. Yet, in changing and unexpected situations (e.g., pandemics, human conflict, natural disasters), specialization trades-off against the need for teams to retain a high capacity to adapt. This inherent compromise raises the question: How to optimize the trade-off, such that health care teams benefit from the safe, repeatable, high-performance of specialization, yet retain a residual capacity for adaptability in the face of acute changes and sudden demands?

Some insight may be gained by analogy to industrial systems that similarly need to optimize trade-offs. The aviation industry, for example, tends to define scopes of practice quite strictly and puts an overarching focus on safety that in effect keeps the level of job plasticity very low. While pilots, cabin crew and flight engineers are all responsible for the successful and safe operation of an aircraft, their roles are only interchangeable under very specific and (hopefully) very rare circumstances. To minimize accidents, these circumstances are delineated by well-defined standards and protocols. A similar focus on accident prevention and safe outcomes has prompted healthcare to embrace a culture of accreditation, protocols, guidelines and the firm assignment of professional roles [17]. The aviation analogy has been useful because it has offered language and frameworks for healthcare to promote a culture of safety. But the analogy breaks down when conditions differ from those prescribed by industry guidelines or demand out-of-the-norm creativity and flexibility. And these conditions are exacerbated by the fact that unlike airplanes, patients are alive. Patients are vastly more complex than even the most sophisticated aircraft. They are also culturally variable and otherwise individualistic. The aviation analogy may thus hamper the development of resilience of healthcare teams if it is adopted too easily. This is where the plasticity principle from the sociobiology analogy may become a useful construct.

**Figure 1:**
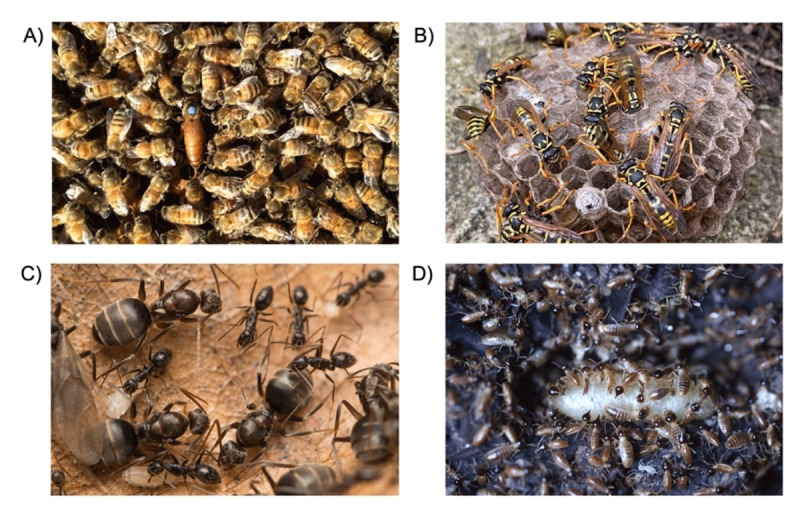
Social insects as models for adaptation of health care teams. Some species of bees **(A)**, wasps **(B)** and all species of ants **(C)** are eusocial, and thus operate in groups that use behavioural specialization and division of labour to complete tasks as a team. Termites **(D)** likewise have evolved castes that each perform certain roles over others. In this photo, the queen is attended by a collection of workers and soldiers (dark heads). Ideally the type, number and ratio of different castes (and subcastes) is well matched to colony need and environmental demand, but where it isn’t, colonies can to some extent interchange the role of task specialists. For example, in some species reproductive specialists (queens; as in A with blue mark, surrounded by workers) can perform worker duties and likewise workers can under certain circumstance lay eggs. We argue that this resiliency to changes in demand can serve as a model for the health care system but would require a similar capacity for role redundancy and adaptability over ridged role assignment. Image credit: A and BEmma Walters, C- François Brassard, D- David Mora.

We suggest that plasticity help us relate the collective behaviour of social insects within colonies (Figure 1) to the collective behaviour of professionals within health care teams. In both cases, individuals respond in some degree to each other’s actions, such that there is a strong social interaction effect that changes in response to the situation itself. Furthermore, the role of the individual is specialized to perform certain tasks that collectively serve a common purpose [18]. While the currency of this purpose is vastly different between systems (Darwinian fitness in social insects vs. patient outcomes in health care), there are organizational parallels [19]. Specifically, the tightly coordinated yet flexible *division of labour* seen in social insect colonies is functionally equivalent to *scopes of practice* that in effect coordinates individual effort into a highly integrated team response.

The analogy between health care teams and social insect colonies is therefore essentially an analogy between these two concept terms. In honey bee colonies, the mated queen single-handedly takes the role of matriarch and principal egg-layer but does little else. Her worker daughters, by contrast, have, in essence, a complementary role: they care for the queen’s brood, forage for pollen and nectar or clean and disinfect the hive, among other highly specialised tasks in response to the needs of the colony. Thus, the queen caste is specialized for reproduction while the worker caste is specialized toward non-reproductive labour (‘reproductive division of labour’) [20]. The worker caste in some highly social species may be further specialised yet into subcastes that each have even more specialized non-reproductive roles. Natural selection can therefore promote the evolution of subdivisions in labour towards evermore specialized yet complementary roles [21]. By comparison in health care teams, we can imagine the one or few doctors to take the role of the queen bee. They perform the main function of the system – for instance, a surgical operation (or equivalent) yet can only do so in the supportive context of the ‘hive’, which consists of a larger number of specialized ‘workers’ that perform all the non-surgical tasks from set-up, to anesthetics, to support, etc. Likewise, the support specialists of health care teams can sub-specialize into ‘subcastes’ as needed.

Despite the adaptive value of divisions of labour and scopes of practice, they can each ironically become maladaptive if the roles become too rigidly specialized to allow for adjustments as might occasionally be needed. This occasional but very important need for flexible specialists is an oxymoronic problem that is not easily solved. Social insects offer one potential solution, namely plasticity: their division of labour is highly specialized yet at once can be flexible such that it is immediately responsive to change in demand. That is, the specialized roles of individual workers within a bee hive or other social insect colony are to some degree interchangeable [22]. Even in honey bee societies with recognizable specialized jobs within the worker caste (e.g., nurses, foragers, guards, hygienists, undertakers, scouts, etc.), an individual’s role can interchange within another, depending on circumstance [22]. For example, if foragers or guards become few in relation to demand, nurse bees can accelerate their behavioural development to forage or guard precociously. That is, the team, in effect, exhibits resilience [23].

## Illustrating plasticity

The use of sociobiology principles remains scarce in healthcare teamwork literature. While some reviews and commentaries make use of the sociobiology language to refer to leadership during crisis [24,25], or report on social insect-inspired algorithms in health care innovation [26,27,28], very little empirical work has been done around the use of sociobiology principles, such as plasticity – expanding one’s task repertoire to take-on another’s when they are unable – to help teams navigate the safety and resilience trade-off. This relationship is at present perceived as a rigid trade-off [29], where aiming to maximize safety and resilience simultaneously has been portrayed as an insurmountable challenge [30]. However, if instead, we aim to master Amalberti’s *art of compromise* [29], opportunities rather than challenges might prevail. For instance, while swapping tasks (or roles or people) may really be unnecessary or unsafe in stable situations, it may nonetheless be necessary when human or technical resources are low or when health care environments are otherwise chaotic. To provide necessary evidence, our empirical work is beginning to offer some insights. For instance, we found that healthcare teams who embraced redeployment (a form of plasticity as task swapping) better managed the various forms of dissonance they experienced at the onset of the Covid-19 pandemic [31]. Overall, we found that teams that value the ability to: a) to read each other’s cues and miscues, b) to step aside when others had the necessary skills, even if outside their scope, c) to deviate from protocols, and d) to foster cross-training adopted an attitude of openness towards plasticity. This attitude allowed them to shift their debriefing conversations from judging what went wrong when they swapped tasks to considering how to best leverage plasticity for success [32]. In our view, team strategies that value a built-in capacity for plasticity, as espoused by the sociobiology analogy will be better situated to navigate the safety and resilience trade-off.

Our argument is that safety and resilience can co-exist in healthcare teams if we think about analogies for integration, not for exclusion. While aviation offers a useful analogy to operationalize the safety mandate, social insects help us to appreciate and understand the equal importance of resilience. Neither analogy offers an ideal model for the behaviour of health care teams but in context each has its merits. To illustrate, in Box 1 we consider a two-by-two contrast in healthcare environments, which create four hypothetical case studies that may differ in plasticity needs. Specifically, we consider community (or rural) hospitals and academic hospitals, which may or may not favour plasticity. We then situate these work settings in stable (or predictable) environments and chaotic (or unpredictable) environments.

**Box 1: The plasticity problem in health care on a case-by-case basis. The capacity of each healthcare setting, as conveyed by hospital type, and the stability of the environment that it serves, are just two variables that can affect decisions on optimal team composition, from a rigid protocol-driven team of single-role members to a team of multi-role players**

**Table:** 

	**Stable/predictable (Safety + Adaptability)**	**Chaotic/unpredictable (Safety + Adaptability)**
Community/Rural hospital (low resources: personnel, equipment)	**Case A** Physicians and nurses at a community or rural hospital performing a routine delivery of a baby, a low-risk colonoscopy, stabilization of car accident injuries that arrive to the emergency room. These are examples of activities that can be successfully performed with a high level of safety even in a low resource setting. Techniques and roles are well-defined and potential risks are predictable and manageable.	**Case C** Emergency room physicians and nurses at a community hospital receiving a patient with internal organs exposed due to a stab wound. The severe injury would ideally be transferred to specialized trauma care and the team has to improvise against guidelines to keep the patient alive while waiting for transfer. Nurses are required to help physicians with tasks outside their prescribed role. The community surgeons conduct trauma-related maneuvers guided by a trauma surgeon from the academic hospital who communicates with them over the phone. Even though there are major threats to patient safety, such interchangeability and adaptation gave the patient a lasting chance.
Academic hospital (high resources: personnel, equipment)	**Case B** Surgical team performing a colon cancer procedure at an academic hospital. Despite the higher level of acuity, risks are predictable, and safety is maintained given the high resource setting where specialized tasks are performed by specialized individuals following established guidelines.	**Case D** During the Covid-19 pandemic services were reorganized and many healthcare providers were assigned to tasks outside their professional comfort zone. For example, operating room nurses and pediatric intensivist physicians were assigned to adult intensive care units. Junior residents were required to do tasks of senior residents. Paramedic, ER physicians, and ER nurses rearranged themselves in small resuscitation teams. It was assumed that while these individuals did not have the full expertise, they could transfer their basic skills in an on-demand manner.

Cases A and B maximize health outcomes by adhering to safety guidelines to prevent harmful improvisation when adaptation is not needed, as per the aviation analogy. However, as Cases C and D show, in chaotic or unprecedented situations, outcomes may well be worst of all, if not for the adaptation enabled by the improvised departure from guidelines. Examples of Case C do not only encompass life and death situations. It could include, say, a sudden food poisoning of the physician of a community hospital when the emergency room is overwhelmed, requiring nurses to spontaneously adopt ‘doctor tasks’. While not complying fully with scope of practice guidelines, this seemingly ill-advised adaptations might, paradoxically, be the difference between good and poor patient outcomes. Therefore, these forms of plasticity can increase effectiveness and efficiency of task performance or increase the speed of response.

## Implications for training

While plasticity has naturally evolved in social insects, infusing plasticity in healthcare teams requires intentional training. Unfortunately, intentional training of on-the-ground healthcare practitioners is plagued by a multitude of challenges. For instance, the heavy workload that leaves little time for on-the-job training, if any; the changing membership of teams that compromises cross-training; or the value we place on specialization over generalism that muddy plasticity efforts [15,33]. However, if disruptive events are here to stay (case in point, the pandemic) and if the current conditions of the system are not ideal for fully embracing plasticity, then maybe this is the time to start pondering about creative ways in which we can strategically infuse plasticity in our healthcare teams to leverage their capacity for resilient teaming. For instance, many have demonstrated the benefits of debriefing in team training, including improved clinical outcomes, team performance and identification of errors [34,35,36]. While debriefing could be a powerful strategy for infusing plasticity, evidence shows that it is still an underused team training strategy [37]. If we are to capitalize on debriefing for infusing plasticity, we suggest that teams should engage in conversations around questions such as: what did this situation call for, generalist or a specialist?, what beliefs guided team members to help outside their specialized role or not?, were team members aware of who to approach who may have the experience they don’t have to take over or to teach them? Therefore, when debriefing for plasticity, instead of dismissing role changes as the best of a bad situation, they can be thought of as an adaptive response that can be productively trained for certain cases. Training for plasticity in healthcare teams may, as has naturally evolved in social insects, increase the team’s behavioural flexibility and boost resilience at the team level.

## Conclusion

Resilient healthcare teams are the backbone of resilient healthcare systems. As modern healthcare systems increase in complexity and decrease in stability, healthcare teams are required to successfully perform in both stable and disruptive situations. However, strategies to train for this goal have only shown minimal effectiveness. A major part of the issue relates to the analogies we use to inspire our thinking. In this paper we offered the sociobiology analogy to help us consider alternative ways to enhance resilience in healthcare teams. In using this analogy in our empirical work, we have learned a key lesson: despite healthcare system’s tremendous emphasis on role specialization and protocols, successful healthcare teams understand that when disruption happens, their ability to remain resilient rests on their ability to change in response to demand, even if that demand is outside of established protocol. We know that when circumstances are predictable and resources abound, the aviation analogy offers a useful framework to prioritize safety. However, too strict an adherence to the aviation analogy may thwart a system’s un-tapped capacity for creativity and resilience. Here is where using analogies in a complementary fashion may prove useful. In this paper, we suggested social insect behaviour as a novel biological analogy to accommodate the realities of healthcare that are not adequately covered by human industry-based analogies. As the mechanisms that enable safety and resilience are distinct, our message is not one of exclusivity. Both analogies should apply, if we are to truly cultivate and effectively train resilient healthcare teams that enable healthcare systems to achieve their intended purpose: ensuring longer, healthy, quality life.

## References

[1] Holldobler B, Wilson EO. The superorganism: the beauty elegance and strangeness of insect societies. WW Norton & Company; 2009.

[2] Bourke AFG. Principles of Social Evolution. New York: Oxford University Press; 2011. DOI: 10.1093/acprof:oso/9780199231157.001.0001

[3] Veen M, Skelton J, de la Croix A. Knowledge, skills and beetles: respecting the privacy of private experiences in medical education. Perspectives on Medical Education. 2020; 9: 111–116. DOI: 10.1007/S40037-020-00565-5 32026318 PMC7138766

[4] Veen M, de la Croix A. How to Grow a Professional Identity: Philosophical Gardening in the Field of Medical Education. Perspectives on Medical Education. 2023; 12: 12. DOI: 10.5334/pme.367 36908744 PMC9997106

[5] Gordon S, Mendenhall P, O’Connor BB. Beyond the checklist: what else health care can learn from aviation teamwork and safety. Cornell University Press; 2019. DOI: 10.7591/9780801465789

[6] Walker B, Holling CS, Carpenter SR, Kinzig A. Resilience, adaptability and transformability in social-ecological systems. Ecol Soc. 2004; 9(2): 5. DOI: 10.5751/ES-00650-090205

[7] Gunderson LH. Ecological resilience – in theory and application. Annu Rev Ecol Syst. 2000; 31: 425–39. DOI: 10.1146/annurev.ecolsys.31.1.425

[8] Ellaway RH, Bates J, Teunissen PW. Ecological theories of systems and contextual change in medical education. Medical Education. 2017; 51(12): 1250–1259. DOI: 10.1111/medu.13406 28857233

[9] Cristancho S, Field E, Lingard L, Taylor T, Hibbert K, Thompson G, Hibbert W. Ecological interchangeability: supporting team adaptive expertise in moments of disruption. Advances in Health Sciences. 2022; 1–22. DOI: 10.1007/s10459-022-10160-4 PMC9648894 36357657

[10] Leonhardt SD, Menzel F, Nehring V, Schmitt T. Ecology and evolution of communication in social insects. Cell. 2016; 164: 1277–1287. DOI: 10.1016/j.cell.2016.01.035 26967293

[11] Vander Meer RK, Breed MD, Winston M, Espelie KE. (eds.) Pheromone communication in social insects: ants, wasps, bees, and termites. Boulder, CO: Westview; 1998.

[12] Cristancho S, Field E. Qualitative investigation of trace-based communication: how are traces conceptualised in healthcare teamwork? BMJ Open. 2020; 10. DOI: 10.1136/bmjopen-2020-038406 PMC7643497 33148735

[13] Middleton EJ, Latty T. Resilience in social insect infrastructure systems. Journal of The Royal Society Interface. 2016; 13: 20151022. DOI: 10.1098/rsif.2015.1022 26962030 PMC4843670

[14] Gordon DM. From division of labor to the collective behavior of social insects. Behavioral Ecology and Sociobiology. 2016; 70: 1101–1108. DOI: 10.1007/s00265-015-2045-3 27397966 PMC4917577

[15] Palmer VJ, Naccarella L, Gunn JM. Are you my generalist or the specialist of my care? Journal. 2007; 34: 4–page.

[16] Firn J, Preston N, Walshe C. What are the views of hospital-based generalist palliative care professionals on what facilitates or hinders collaboration with in-patient specialist palliative care teams? A systematically constructed narrative synthesis. Palliat Med. 2016; 30(3): 240–56. DOI: 10.1177/0269216315615483 26873984

[17] Nelson S, Turnbull J, Bainbridge L, Caulfield T, Hudon G, Kendel D, et al. Optimizing Scopes of Practice: New Models of care for a health care system [Internet]. 2014; p. 44. Available from: https://books-scholarsportal-info.proxy1.lib.uwo.ca/uri/ebooks/ebooks0/gibson_cppc-chrc/2014-11-25/1/10943321.

[18] Seeley TD. The Wisdom of the Hive: The Social Physiology of Honey Bee Colonies. Cambridge, Massachusetts: Harvard University Press; 1995. DOI: 10.4159/9780674043404

[19] Holbrook CT, Clark RM, Moore D, Overson RP, Penick CA, Smith AA. Social insects inspire human design. Biol Lett [Internet]. 2010 Aug 23 [cited 2021 Nov 19]; 6(4): 431–3. DOI: 10.1098/rsbl.2010.0270 20392721 PMC3226954

[20] Wilson EO. 1971. The Insect Societies. Cambridge: Belknap Harvard University Press.

[21] Thompson GJ, Chernyshova AM. Caste Differentiation: Genetic and Epigenetic Factors. In: Encyclopedia of Social Insects, (Starr, C., ed.). 2021; pp. 165–176. Cham, Switzerland: Springer. DOI: 10.1007/978-3-030-28102-1_178

[22] Johnson BR. Organization of work in the honeybee: a compromise between division of labour and behavioural flexibility. Proc R Soc Lond B Biol Sci [Internet]. 2003 Jan 22 [cited 2021 Nov 19]; 270(1511): 147–52. DOI: 10.1098/rspb.2002.2207 PMC1691230 12590752

[23] Cristancho SM. On collective self-healing and traces: How can swarm intelligence help us think differently about team adaptation? Med Educ. 2021; 55: 441–7. DOI: 10.1111/medu.14358 32815185

[24] Marcus LJ, McNulty E, Dorn BC, Goralnick E. Crisis meta-leadership lessons from the Boston marathon bombings response: the ingenuity of swarm intelligence. National Preparedness Leadership Initiative; 2014 Apr 7.

[25] Kardong-Edgren S. Reframing team training as meta-leadership and swarm intelligence. Clinical Simulation in Nursing. 2015 May 1; 11: 276–7. DOI: 10.1016/j.ecns.2015.02.006

[26] Trivedi S, Patel N. The Impact of Artificial Intelligence Integration on Minimizing Patient Wait Time in Hospitals. Researchberg Review of Science and Technology. 2020 Mar 11; 3: 21–35.

[27] Lauritsen SM, Kristensen M, Olsen MV, Larsen MS, Lauritsen KM, Jørgensen MJ, Lange J, Thiesson B. Explainable artificial intelligence model to predict acute critical illness from electronic health records. Nature Communications. 2020; 11: 1–11. DOI: 10.1038/s41467-020-17431-x PMC7395744 32737308

[28] Kulkarni A, Saraf C. Learning from Nature: Applications of Biomimicry in Technology. In 2019 IEEE Pune Section International Conference (PuneCon); 2019 Dec 18 (pp. 1–6). IEEE. DOI: 10.1109/PuneCon46936.2019.9105797

[29] Amalberti R. Resilience and safety in health care: Marriage or divorce? In Resilient Health Care. 2019 Jul 23; 27–38. CRC Press.

[30] Greenhalgh T, Papoutsi C. Studying complexity in health services research: desperately seeking an overdue paradigm shift. BMC Med. 2018 Jun 20; 16(1): 95. DOI: 10.1186/s12916-018-1089-4 29921272 PMC6009054

[31] Cristancho S, Field E, Taylor TS. Adapting despite “walls coming down”: Healthcare providers’ experiences of Covid-19 as an implosive adaptation. Perspect Med Educ. 2022; 1–7. DOI: 10.21203/rs.3.rs-953055/v1 35635718 PMC9150045

[32] Cristancho S, Field E, Bader-Larsen KS, Varpio L. Interchangeability in military interprofessional health care teams: Lessons into collective self-healing and the benefits thereof. Military Medicine. 2021; 186(Supplement_3): 16–22. DOI: 10.1093/milmed/usab122 34724051

[33] Dower C, Moore J, Langelier M. It is time to restructure health professions scope-of-practice regulations to remove barriers to care. Health Affairs. 2013; 32(11): 1971–1976. DOI: 10.1377/hlthaff.2013.0537 24191088

[34] Wolfe H, Zebuhr C, Topjian AA, et al. Interdisciplinary ICU cardiac arrest debriefing improves survival outcomes. Crit Care Med. 2014; 42(7): 1688–1695. DOI: 10.1097/CCM.0000000000000327 24717462 PMC4092119

[35] Hoyt DB, Shackford SR, Fridland PH, et al. Video recording trauma resuscitations: an effective teaching technique. J Trauma. 1988; 28(4): 435–440. DOI: 10.1097/00005373-198804000-00003 3352005

[36] Bandari J, Schumacher K, Simon M, et al. Surfacing safety hazards using standardized operating room briefings and debriefings at a large regional medical center. Jt Comm J Qual Patient Saf. 2012; 38(4): 154–160. DOI: 10.1016/S1553-7250(12)38020-3 22533127

[37] Sandhu N, Eppich W, Mikrogianakis A, Grant V, Robinson T, Cheng A. Postresuscitation debriefing in the pediatric emergency department: a national needs assessment. CJEM. 2013; 15: 1–10. 25227647

